# High prevalence of diabetic neuropathy in academic outpatient league for diabetes at University of Uberaba

**DOI:** 10.1186/1758-5996-7-S1-A5

**Published:** 2015-11-11

**Authors:** Fernanda Oliveira Magalhães, Eduarda Martins Medeiros, Luciana Souza Lima, Patricia Ibler Bernardo Ceron, Isabel Cristina Resende Lopes, Victor Peixoto de Almeida, Duanny Lorena B M Caetano, Marcela Sousa Bernardes, Jéssica Staciarini Ponciano Silva, Douglas Donizeth Rezende, Rodrigo Ribeiro

**Affiliations:** 1 Universidade de Uberaba, Uberaba, Brazil

## Background

“Diabetic Foot” is the infection, ulceration and/or destruction of the deep tissues associated with neurological abnormalities and various degrees of peripheral vascular disease in the lower limb in diabetic patients.

## Objectives

To identify the prevalence of neuropathy and diabetic vasculopathy in patients treated in the outpatient clinic of the League of Diabetes.

## Materials and methods

The League of Diabetes adopted screening for diabetic foot, recommended by the Brazilian Society of Diabetes and BrasPEDI-Brazilian Group of Diabetic Foot, through the achievement of a score of neuropathic symptoms and signs (sensitivities tactile, painful, vibration, thermal and Achilles reflex), and clinical evaluation of arteriopathy. The work was carried out by members of the League of Diabetes of the Medical Course, in all patients treated during the year 2014, under supervision. The data were represented as percentages, and analyzed by using SPSS 14.0, through the Chi-square test, with a significance level of 5%.

## Results

Among the collected data, a total of 73 diabetic patients analyzed, 67.1% had neuropathy, 32.9% arteriopathy of the lower limbs, 9% previous ulcer, 1.4% active ulcer and 1.4% lower limb amputation. For neuropathy, 15.1% presented with diabetic polyneuropathy (DPN) painful, 24.7% painful polyneuropathy with risk of ulceration, 4.1% DPN asymptomatic, 23.3% neuropathic pain and 32.9% without neuropathy. There was association of diabetic neuropathy with: time of diabetes (Chi2=7.789, p=0.020), being more frequent in individuals with 6-15 yrs. of disease; retinopathy (Chi2=3.733, p=0.05); age (Chi2=10.979, p=0.027) was more frequent after 51 years of age; and presence of vasculopathy (Chi2=13.627, p< 0.001) where 95.7% of the individuals with vasculopathy also have neuropathy. The single person with lower limb amputation was also a carrier of diabetic neuropathy.

## Conclusion

The thorough examination and the early approach of diabetic patients are essential to enable develop efficient actions and thus, intervene early, in addition to promoting a better prognosis for the patient, raising their quality of life. Diabetic neuropathy is a complication highly prevalent and disabling, and should be diagnosed and treated early in all diabetic patients.

